# European catfish (*Silurus glanis*) as a freshwater apex predator drives ecosystem via its diet adaptability

**DOI:** 10.1038/s41598-017-16169-9

**Published:** 2017-11-21

**Authors:** Lukáš Vejřík, Ivana Vejříková, Petr Blabolil, Antti P. Eloranta, Luboš Kočvara, Jiří Peterka, Zuzana Sajdlová, Son Hoang The Chung, Marek Šmejkal, Mikko Kiljunen, Martin Čech

**Affiliations:** 1 Biology Centre of the Czech Academy of Sciences, Institute of Hydrobiology, Na Sádkách 7, 37005 České Budějovice, Czech Republic; 2 Faculty of Science, University of South Bohemia in České Budějovice, Branišovská 31, 37005 České Budějovice, Czech Republic; 3 Norwegian Institute for Nature Research, P.O. Box 5685 Sluppen, NO-7485 Trondheim, Norway; 4 University of Jyväskylä, Department of Biological and Environmental Science, P.O. Box 35, FI-40014 University of Jyväskylä, Jyväskylä, Finland

## Abstract

Apex predators play a key role in ecosystem stability across environments but their numbers in general are decreasing. By contrast, European catfish (*Silurus glanis*), the European freshwater apex predator, is on the increase. However, studies concerning apex predators in freshwaters are scarce in comparison to those in terrestrial and marine ecosystems. The present study combines stomach content and stable isotope analyses with diet preferences of catfish to reveal its impact on the ecosystem since stocking. Catfish niche width is extremely wide in comparison to the typical model predator, Northern pike (*Esox lucius*). Catfish and pike have different individual dietary specialization that results in different functional roles in coupling or compartmentalizing distinct food webs. The role of both species in the ecosystem is irreplaceable due to multiple predator effects. The impact of catfish is apparent across the entire aquatic ecosystem, but herbivores are the most affected ecological group. The key feature of catfish, and probably a common feature of apex predators in general, is utilization of several dietary strategies by individuals within a population: long-term generalism or specialization and also short-term specialization. Catfish, similar to other large-bodied apex predators, have two typical features: enormous generalism and adaptability to new prey sources.

## Introduction

Large-bodied apex predators play a key role in community dynamics and ecosystem stability^1–3^ due to their generalist foraging strategy on prey at different trophic levels and from different habitats^4–7^. In essence, these consumers tend to incorporate energy from a wide range of prey taxa and thereby often link multiple energetic pathways^8–10^.

The difference among particular species of apex predators is whether all individuals are true generalists or whether they form specialized subpopulations^5,11^ or even display individual niche specialization (INS)^12^. The wide diet plasticity of apex predators is driven by the requirement to satiate their large body and also by their ability to learn to utilize new food sources^13^.

Therefore, the niche width of each apex predator is extremely broad^3,4^. Generalism of apex predators far exceeds dietary habits of other mesopredators in terrestrial^4^ and marine ecosystems^6,14^. Thus the presence of apex predators influences lower situated members of food webs including mesopredators^3,7,15^. Decline or disappearance of an apex predator in an ecosystem causes a cascade effect of changes^2,16,17^. E.g. mesopredators can step in to the role of apex predator^15^. Nevertheless, a mesopredator has size and hunting limitations in comparison to an apex predator, and can therefore have a negative impact on ecosystem stability^18^. Therefore, understanding the role and potential impacts of apex predators is essential for management and protection of freshwaters that provide vital ecosystem services and contribute disproportionally to the global biodiversity^19^. Recently, numbers of apex predators have decreased in terrestrial^1,3^, marine^16,20,21^ and also in freshwater ecosystems^7,19,22^. The impact of the decline of apex predators is a topical ecological problem and has been widely studied^3^. However, comprehensive studies concerning the changes in freshwater ecosystems caused by the decline of apex predators are lacking. Global triggers inducing the trend of decline are climate changes^20^, overexploitation and/or other anthropogenic impacts^3,21^. Contrary to general apex predator decline, European catfish (*Silurus glanis*) metapopulation size and distribution range has increased in recent decades^23^. It is the largest freshwater fish in Europe and the third largest in the world (it reaches 2.7 m and 130 kg)^22,23^. The increase in number and dispersion to new localities is mainly induced by human activity^23–25^. Studies about European catfish focus mainly on diet specifications^26–28^ or they are regional and only describe one characteristic (see review^23^). But the question about their role in an ecosystem, whether it is a key apex predator or not, has not been addressed yet. The main reason for the absence of studies dealing with European catfish is due to difficulties connected with capturing of this fish^29^. Due to the scarcity of information concerning this species, several myths about its unprecedented appetite have appeared among anglers and even among scientists^30,31^.

The main goal of this study was to reveal whether catfish represents a true apex predator with some features known from terrestrial and marine ecosystems in spite of the structural and functional differences between these ecosystems and between life cycles of their species^3,10,15^. Attention has been paid whether it is a generalist species with broad dietary strategies, that stands above other members of the food web. We compared European catfish with Northern pike (*Esox lucius*), as it is a well-studied freshwater fish species and it is often used as a model predator in studies from freshwaters^32^. Pike is an ideal reference species because it is a high-level freshwater predator, the second biggest fish predator in Europe (it reaches 130 cm and over 25 kg), and it also has the widest extent of distribution in freshwaters^33^.

The second goal was to use stable isotope analysis (SIA) of recaptured individuals and stomach content analysis of European catfish and Northern pike to fully understand dietary strategies and degree of specialization of these two freshwater predators. We investigated whether they exhibit individual trophic specialization and whether it is a short-term (seasonal) or a long-term specialization^10^. We focused on the total niche width of European catfish and Northern pike, and on the food origin, whether they utilize food sources only from the aquatic food web^7,35,36^.

Last goal was to assess the impact of apex predators on lower-level members of the food web based on their diet preferences and long-lasting monitoring of fish communities in two study lakes and in the reference lake with similar fish community including pike but with absence of catfish in the system.

## Results

### Characterization of food webs, niche width and patterns of individual specialization

**Figure 1:**
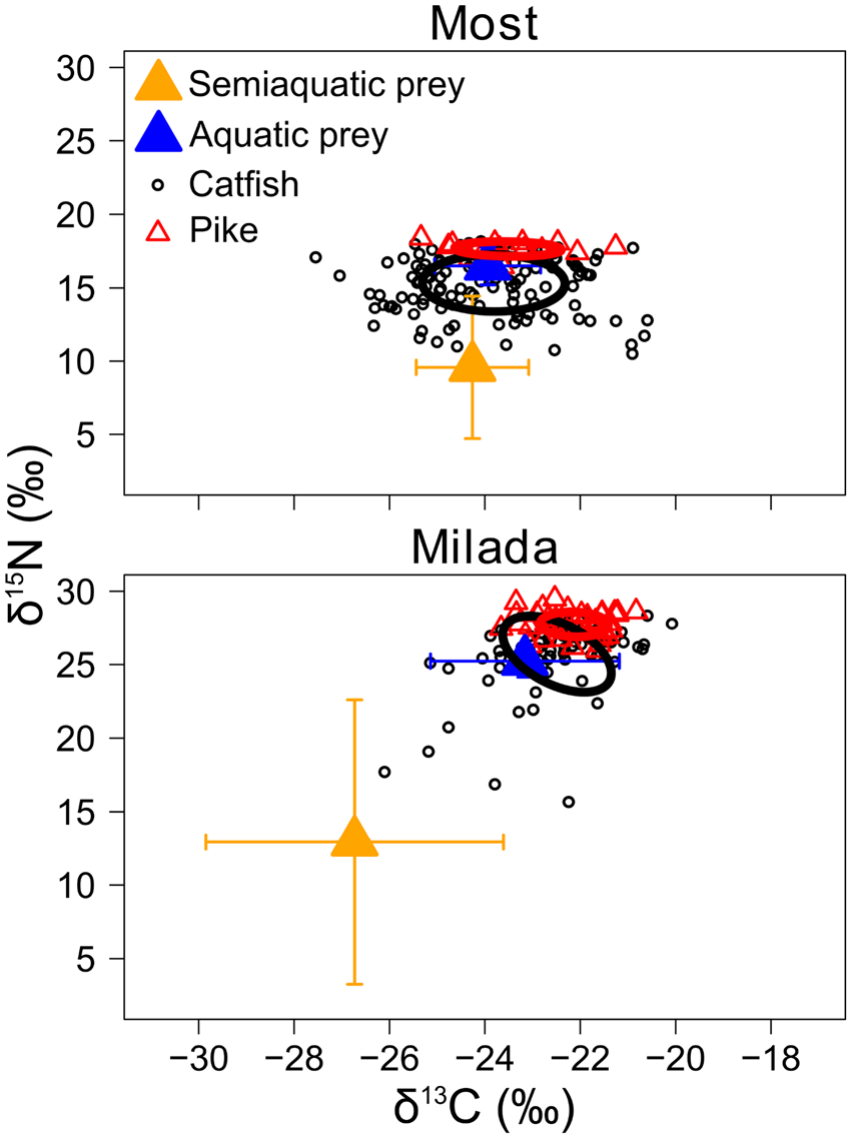
The δ^13^C and δ^15^N values of individual fish (muscle tissue) and the estimated isotopic niches of catfish (black) and pike (red) in Milada and Most lakes, illustrated as sample-size-corrected *SEA*c ellipse areas^36^. The mean ± SD δ^13^C and δ^15^N values of putative semiaquatic and aquatic food resources are also shown.

**Figure 2:**
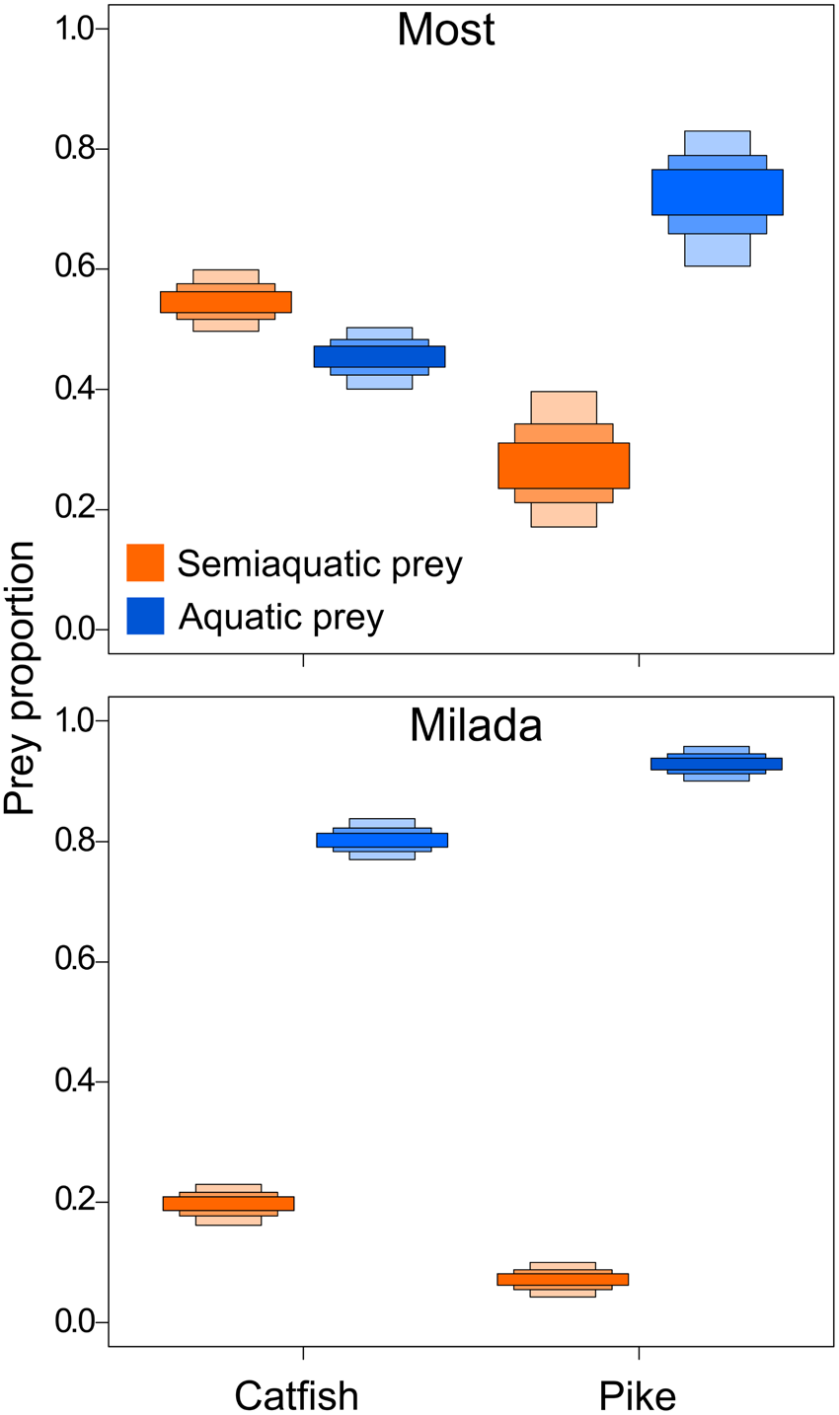
Relative contribution of semiaquatic and aquatic food resources in the long-term diets of catfish and pike in Most and Milada lakes. The boxes indicate the 95, 75 and 50% Bayesian credibility intervals for estimates based on SIAR model (Stable Isotope Analysis in R; version 4.2;^36^) isotopic mixing model.

In both lakes, the semiaquatic vertebrates were isotopically distinct from aquatic food sources due to lower δ15 N values (Fig. 1). However, differences in isotopic signatures of the semiaquatic and aquatic prey in Most Lake were smaller (“average differences here for both isotopes”) than in Milada Lake (“average differences here for both isotopes”) (Fig. 1), probably resulting SIAR (Stable Isotope Analysis in R) results for Most Lake to be slightly more diffused in contrast to Milada Lake (Fig. 2). The results from SIAR isotopic mixing model indicated that in both lakes, catfish utilized more semiaquatic vertebrates (mammals, frogs and birds) than pike (Fig. 2). Semiaquatic prey were a particularly important food for catfish in Most Lake (SIAR 95% credibility intervals: 50–61%) and to lesser extent in Milada Lake (SIAR 95% credibility intervals: 18–23%). In contrast, SIAR indicates that semiaquatic prey is not such an important food source for pike. In Most Lake, pike seem to feed on semiaquatic prey in some extent (SIAR 95% credibility intervals: 18–40%), but the contribution of semiaquatic prey is apparently low in Milada Lake (0–10%; Fig. 2).

In both lakes, the SIBER (Stable Isotope Bayesian Ellipses in R) results indicated a markedly (3–11 times) wider long-term dietary niche for catfish compared to pike (Table 1, Fig. 1). The wider isotopic niche of catfish was due to large individual variation in both carbon sources (δ^13^C) and estimated trophic position (δ^15^N). In both lakes, catfish individuals with exceptionally low δ^15^N values were likely specialized on semiaquatic prey, whereas individual fish with high δ^15^N values were probably specialized on piscivorous diets, possibly including also conspecifics.

**Table 1:** Estimated isotopic niche widths of European catfish and pike in Most and Milada lakes, based on SIBER (Stable Isotope Bayesian Ellipses in R) estimates of standard ellipse (*SEA* and *SEA*_c_) and total convex hull (*TA*) areas (for details see^35^). The *SEA*_c_ overlap indicates the proportional overlap between the sample-size corrected ellipse areas and hence the degree of niche segregation between European catfish and pike.

**Lake**	**Species**	***SEA***	***SEA***_**c**_	***TA***	***SEA***_**c**_ **overlap**
Most	Catfish	9.1	9.2	41.9	0.01
Most	Pike	1.7	1.9	3.9	
Milada	Catfish	8.0	8.2	48.9	0.07
Milada	Pike	1.9	2.0	6.9	

The minor (1–7%) overlap between the *SEA*_c_ (corrected standard ellipse areas) isotopic niche areas (Table 1) indicate significant niche segregation between the species, with catfish occupying a “lower trophic position” (due to utilization of semiaquatic vertebrates, on average 2.0–2.3‰ lower δ^15^N) than pike in both lakes (Most: t = −10.6, df = 73.2, *p* < 0.001; Milada: t = −5.9, df = 92.5, *p* < 0.001). No notable differences were found in carbon sources, although catfish had slightly (on average 0.4‰) lower δ^13^C values than pike in Milada Lake (Most: t = −0.9, df = 18.7, *p* = 0.370; Milada: t = −2.0, df = 1105.3, *p* = 0.044).

**Figure 3:**
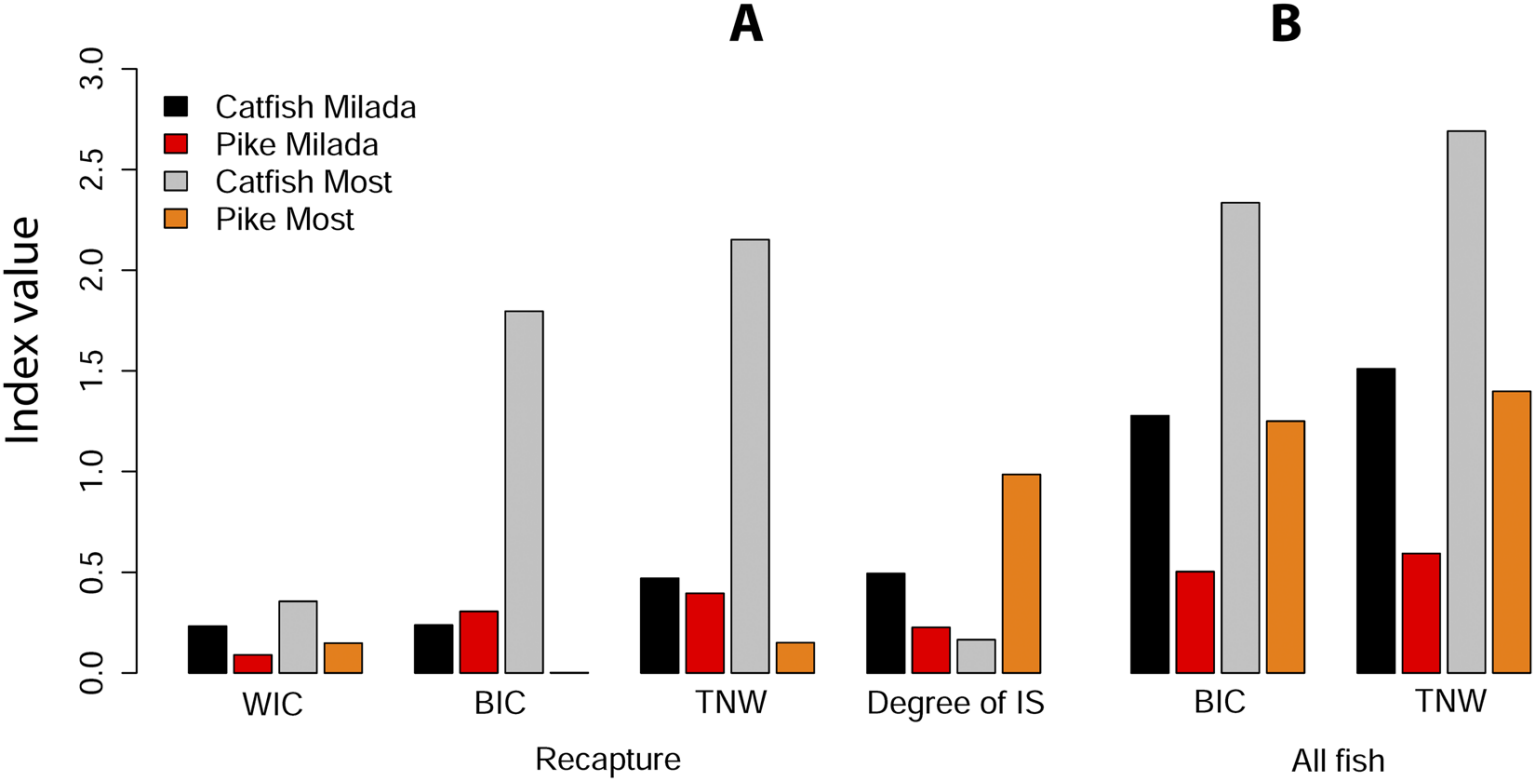
Total niche width (TNW) of catfish and pike population in Most and Milada lakes divided into two components: within-individual component (WIC) and between-individual component (BIC). Degree of individual specialization (IS) ranging from 0 to 1 shows whether each individual in a population utilizes the whole niche width of the population (then IS = 1). The calculations were provided for (**A**) recaptured individuals and for (**B**) all captured individuals.

According to analysis in Ind Spec programme based on δ^13^C, total niche width (TNW) of catfish in both lakes was approximately twice as high as that of pike. Moreover, TNW in Most with less potential food sources was twice as high as that in Milada, when considering all sampled individuals (Fig. 3). Similarly, the within individual component of variation (WIC) of catfish was 2.5 times higher than that of pike in both lakes and also higher in Most than in Milada for both species. The degree of individual specialization (IS) was 0.17 and 0.49 for catfish in Most and Milada Lakes, respectively. In terms of pike, the degree of IS was unexpectedly high in Most (0.98; but the value was probably biased by the low number of recaptures), and half the value in Milada (0.23) in comparison to catfish in Milada (Fig. 3).

### Diet composition according to stomach content analysis and seasonal specialization

The prey fishes found in catfish stomachs included one herbivorous species (rudd: *Scardinius erythrophthalmus*), four omnivorous species (roach *Rutilus rutilus*, tench *Tinca tinca*, ruffe *Gymnocephalus cernuus* and whitefish *Coregonus* sp.), three species that we may call mesopredators (perch *Perca fluviatilis*, asp *Aspius aspius*, and pike) and European catfish itself indicating cannibalism. Among semiaquatic vertebrates, there were found five species of birds (great cormorant *Phalacrocorax carbo*, great crested grebe *Podiceps cristatus*, coot *Fulica atra*, great black-backed gull *Larus marinus*, and reed warbler *Acrocephalus scirpaceus*), two species of amphibians (marsh frog *Rana ridibunda* and edible frog *Rana esculenta*) and one species of mammal living on the shoreline (European water vole *Arvicola terrestris*). Among aquatic invertebrates, there were found spiny-cheek crayfish (*Orconectes limosus*) and larvae of Emperor dragonfly (*Anax imperator*).

**Figure 4:**
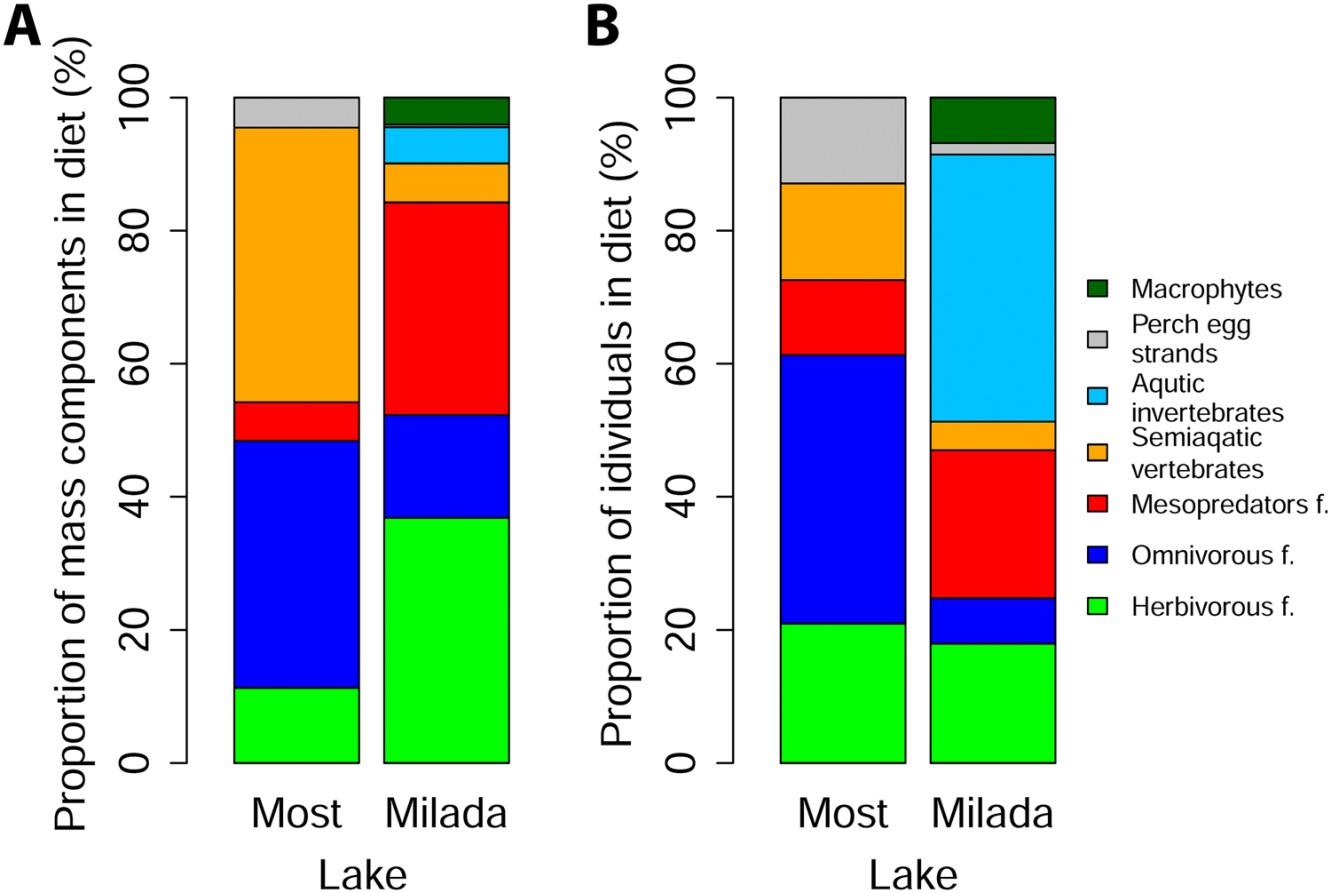
Diet composition of catfish in Most and Milada lakes based on stomach contents divided into seven basic groups of food sources. Prey fish is represented in three groups: herbivorous fish, omnivorous fish and fish mesopredators. Mass ratio and percentage ratio of each food item in the catfish diet are shown in part (**A**) and part (**B**), respectively.

In Most Lake, 72% of catfish had an empty stomach, 10% had our bait and 18% had their actual diet. Altogether, 65 food items were found in the catfish diet from Most Lake. It consisted of seven fish species, five species of waterfowl, one species of aquatic mammal, two species of amphibians, and perch egg strands. In Milada Lake, 40% of catfish had an empty stomach, 6% had our bait and 54% had their actual diet. Altogether, 117 food items were found in the catfish diet from Milada Lake. It consisted of eight fish species, two species of waterfowl, one species of aquatic mammal, two species of invertebrates, perch egg strands, and unidentified macrophytes. The mass ratio and percentage ratio of each food item in catfish diet is shown in Fig. 4.

**Figure 5:**
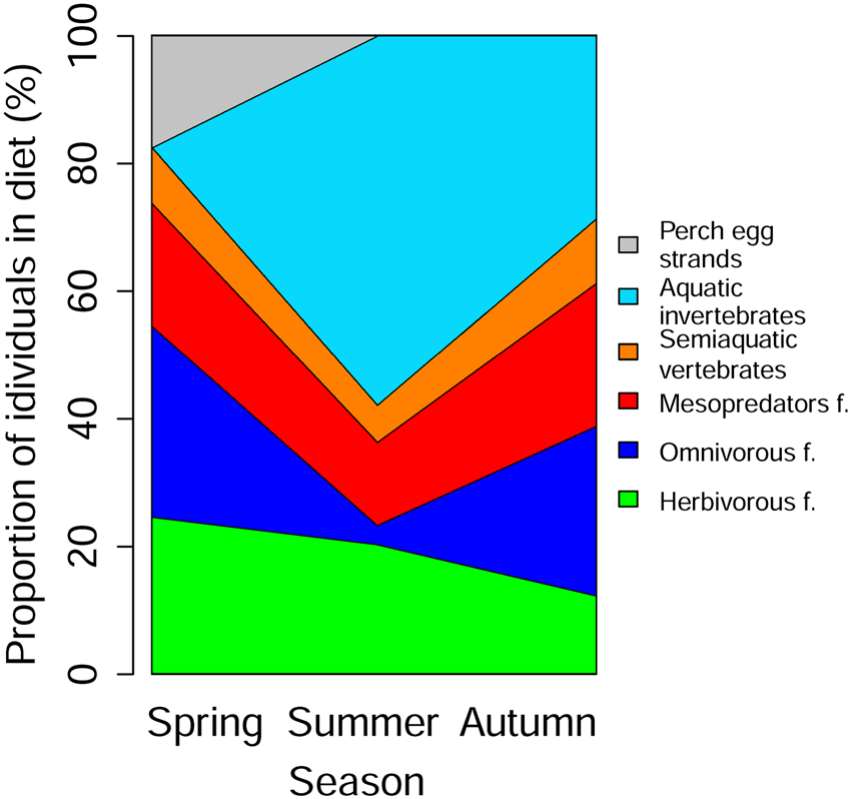
Percentage ratio of each food source found in catfish diet along the seasons pooled from both Most and Milada lakes. Food items found in stomachs were divided into seven basic groups. Macrophytes are not presented due to inability of separation into individuals and due to probable accidental suction with another food items.

Seasonal preferences for certain food sources were observed in catfish diet in both lakes. We found 63, 66 and 53 food items in catfish stomachs during spring, summer and autumn, respectively. Perch egg strands were found only during the perch spawning period in spring (χ^2^ = 36.62, *p* < 0.001). Aquatic invertebrates (crayfish and dragonfly larvae) were fully absent during spring and were present mainly during summer (χ^2^ = 58.20, *p* < 0.001; Fig. 5). In contrast, the contribution of omnivorous fish was the lowest during summer (χ^2^ = 22.13, *p* < 0.001 (Fig. 5) and the highest during spring (corresponding to the roach mass spawning period). Particularly in Most Lake in 2015, roach composed 88% of all food items found in catfish stomachs. Herbivorous fish were preferred particularly in spring and ignored in autumn, but neither was significant (χ^2^ = 4.53, *p* > 0.05). Differences in the contribution of mesopredators (χ^2^ = 2.28, *p* > 0.05) and semiaquatic vertebrates (χ^2^ = 1.05, *p* > 0.05) in catfish diet among seasons were not statistically significant (Fig. 5).

In terms of pike, 7 and 12 food items were found in their stomachs from Most and Milada Lakes, respectively. In Most Lake, we identified roach five times, one perch and one whitefish. In Milada Lake, we identified roach nine times, perch twice and one tench. Evidently, only prey fish was found in pike stomachs.

The prey-to-predator length ratio (PPR) for catfish ranged from 0.04 to 0.51 (mean ± SD: 0.24 ± 0.08) in Most Lake and from 0.05 to 0.57 (mean ± SD: 0.17 ± 0.11) in Milada Lake. The size and mass of prey found in catfish stomach ranged from 4 cm and 0.5 g (larvae of dragonfly) to 68 cm and 2,750 g (asp). PPR for pike ranged from 0.14 to 0.29 (mean ± SD: 0.25 ± 0.07) in Most Lake and from 0.15 to 0.30 (mean ± SD: 0.22 ± 0.04) in Milada Lake (for details see Supplementary Table S1). The size and mass of prey found in pike stomachs ranged from 10 cm and 9.5 g (roach) to 30 cm and 351 g (roach).

### Food preferences and impact on the ecosystem

**Figure 6:**
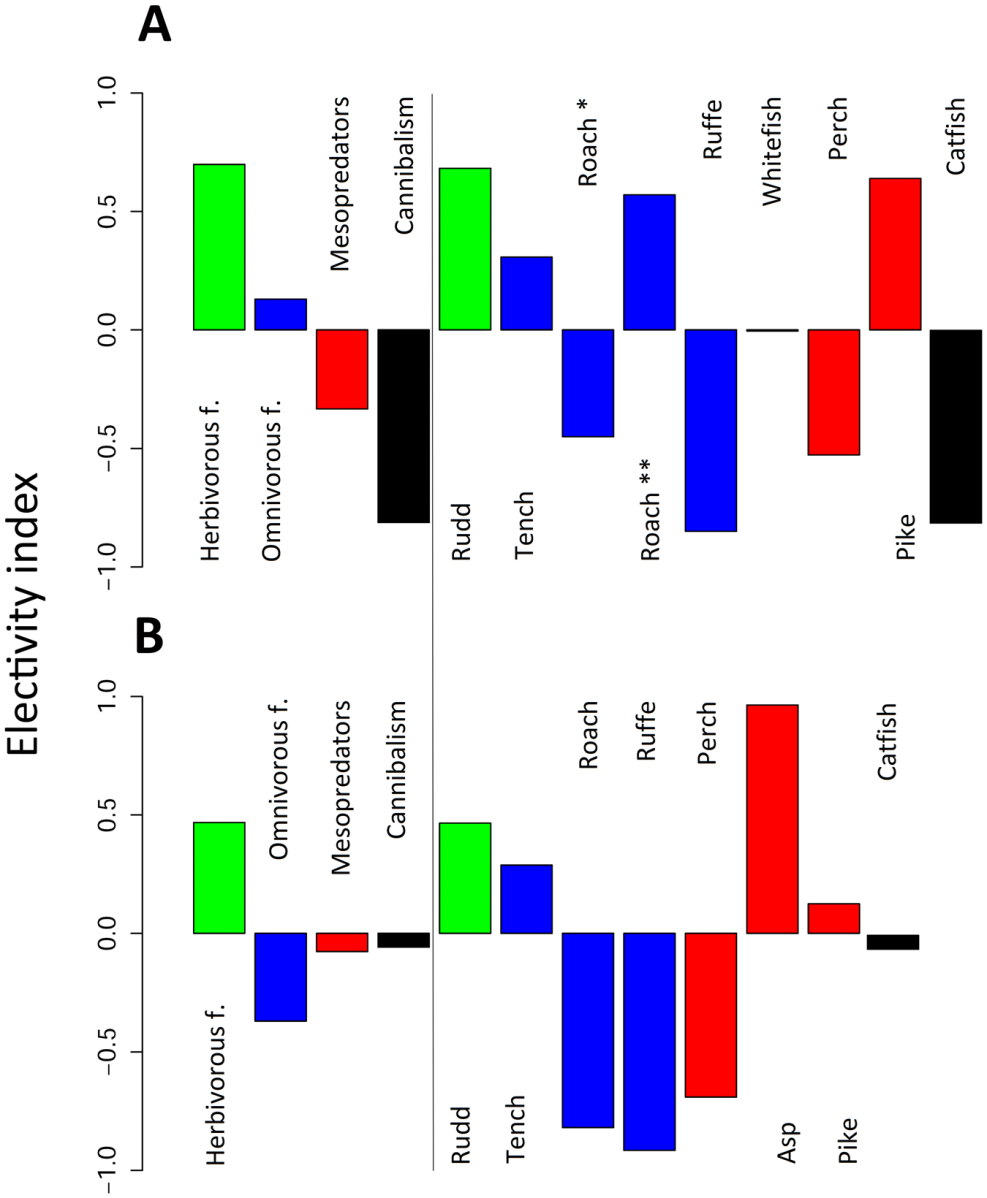
Electivity index of catfish (defined as ratio of relative biomass of a prey in a predator’s diet and relative biomass in the ecosystem based on mean gillnet catches in 2013–2015) for (**A**) Most and (**B**) Milada. Green colour stands for herbivorous fish (only rudd), blue colour for omnivorous fish (tench, roach and ruffe in both lakes and whitefish in Most Lake), red colour for fish mesopredators (perch and pike in both lakes and asp in Milada Lake) and black colour for cannibalism, *i*.*e*., utilizing of catfish. Value 1 responds to full preference and −1 to total ignorance of food item in the diet. One asterisk stands for *E*_*i*_ of roach in Most Lake obtained from seven sampling campaigns except May 2016 during spawning period and two asterisks stand for *E*_*i*_ only from May 2016.

The electivity index (*Ei*) concerning only prey fish in catfish diet revealed marked preferences for herbivorous fish (rudd) in catfish diet. The *Ei* reached 0.68 and 0.47 in Most and Milada, respectively. Positive values of *Ei* were also reached for mesopredators, namely pike, 0.64 and 0.12 in Most and Milada, respectively. Although asp was found only twice in catfish diet in Milada Lake, the *Ei* was the highest (*Ei* = 0.96) due to its low biomass in the lake. A lower *Ei*, but still with positive values, was reached for omnivorous tench, 0.31 and 0.29 in Most and Milada, respectively. Omnivorous roach in Most Lake was the last type of fish with a positive *Ei*, this was mainly due to sampling provided in May 2015 during roach reproduction. If we exclude this sampling campaign, the index is negative (Fig. 6). The *Ei* for other fish was negative in both lakes. Therefore, herbivorous species were in both lakes the most preferred fish out of four fish groups, i.e. herbivores, omnivores, mesopredators and cannibalism (Fig. 6).

Pike markedly preferred roach in the diet. The *Ei* for roach was 0.44 and 0.45 in Most and Milada, respectively. However, the highest index was recorded for whitefish in Most Lake (*Ei* = 0.70). A positive value was recorded also for tench in Milada Lake (*Ei* = 0.12). All three mentioned species were grouped in omnivorous fish, and this group was generally the most preferred food source by pike, the *Ei* reached 0.24 and 0.40 in Most and Milada, respectively. Out of the mesopredators, we found perch in the diet, but the *Ei* was negative, −0.36 and −0.51 in Most and Milada, respectively. Neither herbivorous fish nor European catfish were found in pike stomachs.

**Figure 7:**
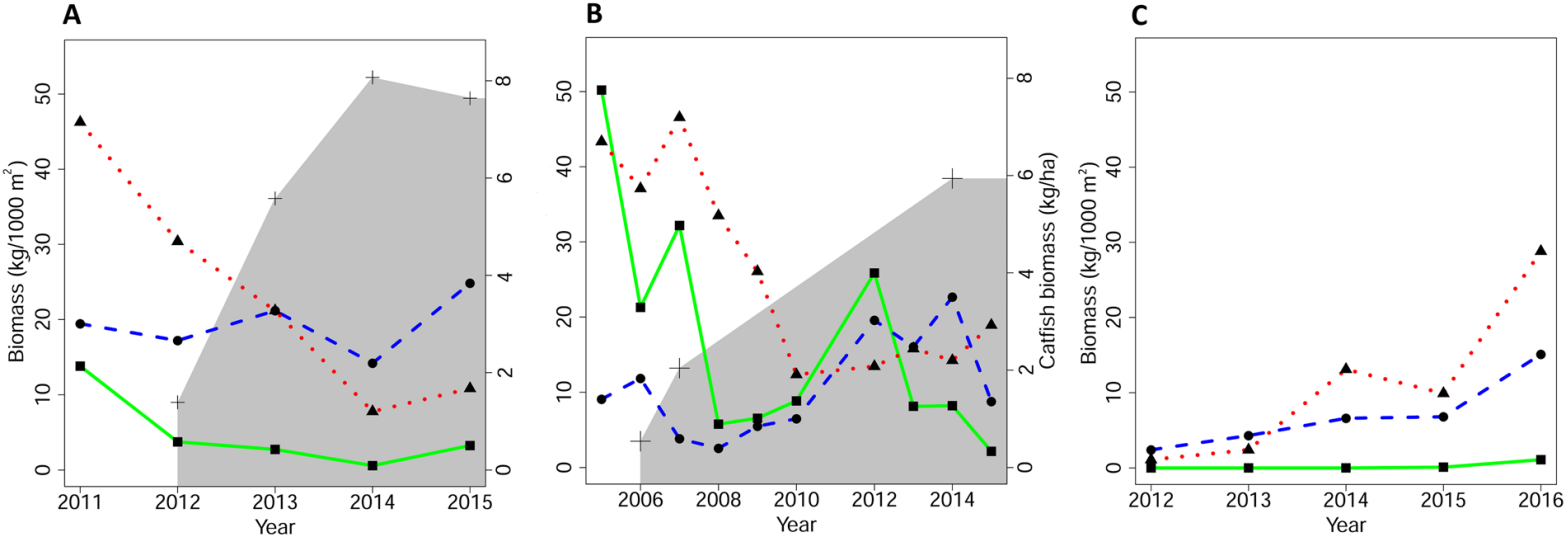
Biomass of three fish groups (herbivores: green, omnivores: blue, mesopredators: red) in gillnet catches (fish older than 0 + ; kg per 1,000 m^2^ of gillnets) of (**A**) Most, (**B**) Milada and (**C**) Medard. Pike was stocked in 2011–2013 (855 kg each year) and 2005 (237 kg) to Most and Milada, respectively. In Medard, pike occurred from the beginning (*i*.*e*. 2008). Beginning of grey part shows the first relevant presence of catfish in the lakes (2012 for Most and 2006 for Milada) and subsequently represents estimated biomass of catfish population (kg ha^−1^). Catfish were stocked in autumn of previous years (2011 for Most and 2005 for Milada), in all cases well after individual gillnet sampling campaigns. This is the reason why illustrations of catfish presence begin one year later, when gillnet catches reflect (for the first time) potential impact of catfish on populations of herbivorous, omnivorous and mesopredatory fishes. Biomass for years 2012–2014 in Most and 2006–2007 in Milada is based on cumulative amount of stocked fish. Biomass for year 2015 in Most and 2014–2015 in Milada is calculated from recaptures.

The fish community has changed dramatically since European catfish and Northern pike were stocked in the lakes (Fig. 7). We recorded lower catches of herbivorous fish and mesopredators by gillnets. In Most Lake, the decrease in herbivores was of 72% during the first year and the abundance of herbivores, compared to the former population, was 16% in the following years. In Milada Lake, the direct decrease of herbivores was 50% and abundance of herbivores was approximately 20% of the former population in the following years. In terms of mesopredators, numbers in Most Lake decreased by 34% and abundance was 29% compared to the former population. In Milada Lake, mesopredators also decreased rapidly and the abundance after five years stabilized at 35% compared to the former population (Fig. 7). In contrast, the population of omnivorous fish did not apparently change. In Most Lake, practically no changes were recorded in the abundance of omnivorous fish in spite of slight fluctuation. In Milada Lake, the abundance of omnivorous fish increased by 17% (Fig. 7). In contrast to study lakes, fish biomass was gradually increasing in the reference Medard Lake with no catfish. Abundance of omnivorous fish and mesopredators increased six and 26 times, respectively. Unfortunately, herbivorous rudd was primarily absent in the reference lake, its presence was evidenced in 2015 (0.1 kg per 1,000 m^2^ of gillnets) and its biomass increased markedly in 2016 (1.1 kg per 1,000 m^2^ of gillnets). However, increase of mesopredators is apparent in Medard in comparison to other two study lakes, exceeding omnivorous fish since 2014 with almost twice as high population in 2016 (mesopredators 28.8 kg and omnivores 15.1 kg per 1,000 m^2^ of gillnets).

According to feed conversion ratio (FCR), catfish population consume annually 528–1,232 kg (1.7–4 kg ha^−1^) and 475–1,109 kg (1.9–4.4 kg ha^−1^) of food in Most and Milada, respectively (considering selected FCR). Detailed specification of food items and their masses consumed annually by catfish is shown in Table 2.

**Table 2:**
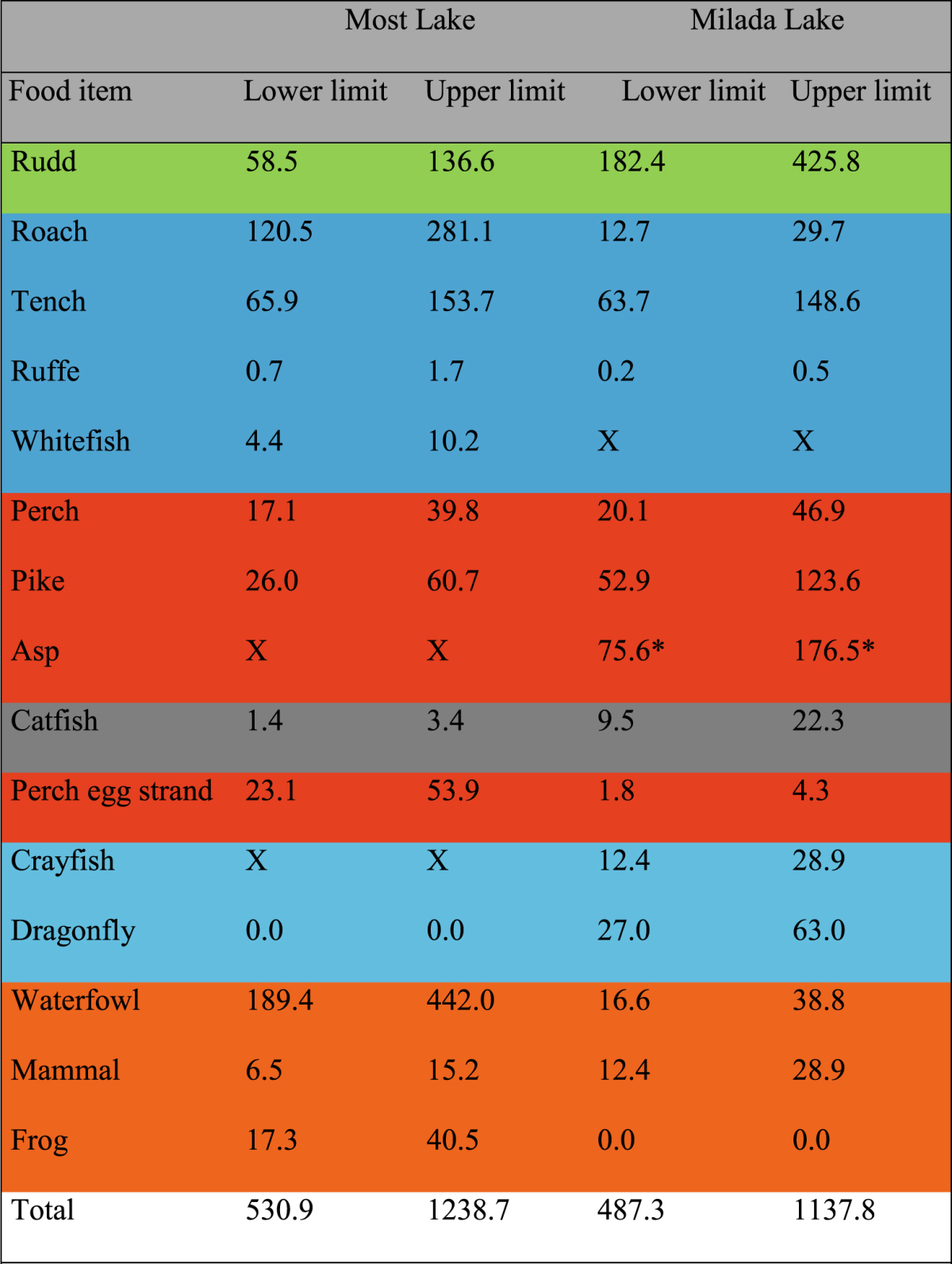
Estimated mass (kg) of each food item consumed annually by catfish population in Most and Milada lakes. Green colour: herbivores, blue: omnivores, red: mesopredators, black: cannibalism, light blue: aquatic invertebrates, orange: semiaquatic vertebrates. X stands for food items that do not occur in the lakes. The estimation marked with asterisk was based only on two observations but with high mass of the prey.

## Discussion

Terrestrial and many marine predators have visual and olfactory prey detection^37^. Visual prey detection is probably crucial even for freshwater predators such as pike and perch^32,34^. In contrast, European catfish have an entirely different method of detection by hydrodynamic traces^27,38^. Thus, catfish more easily detects a moving prey item than a static one. Despite the various hunting strategies of apex predators across a clade, their eventual impact on the ecosystem is very similar. The strategy of catfish naturally influences hunting activity, which takes place predominantly at night^24^. This is supported also by our catches, because the majority of catches (87%) were made during the night. It seems also to be the reason why catfish frequently utilizes prey that are active at night, such as rudd^39^, tench^40^, or crayfish^41^.

In contrast, pike hunts for food mainly during the day, which is reflected in the composition of its diet^32,33,42^. Therefore, interspecific competition for food between catfish and pike is of low relevance. This is apparent also from the stomach contents and minor niche overlap. Hence it seems that an ecosystem can be shared by both predators without major problems. European catfish is a true generalist, but roach is rare in its diet, whereas it is the main food item of pike. A similar situation also occurs in the marine ecosystem, where tiger shark (*Galeocerdo cuvier*) is a generalist, whereas bull shark (*Carcharhinus leucas*) has specialist individuals^10^.

According to high values of δ^15^N, pike seems to be at higher trophic level than catfish. However this is misleading information. Pike is a piscivorous species preferring a limited number of species (mainly roach and usually perch but not in our case) with a tendency to cannibalism^32^. The niche width of pike proves narrow specialization of this species. In contrast, lower values of δ^15^N may seem to indicate a lower trophic level of catfish but the main reason is the utilization of semiaquatic vertebrates with low δ^15^N.

Based on the electivity index of catfish, there is relatively intensive predation on pike. The opposite predation is most probably low. We did not find catfish in any pike stomachs. The reason is probably that pike are active during the day^42^, whereas catfish are active during the night already from small sizes and are hidden in a refuge during the day^24^. This fact puts European catfish in the position of apex predator in freshwater ecosystems. Beside Northern pike, European catfish is also able to utilize other mesopredators, such as perch and asp. Differences in populations of mesopredators between lakes with and without catfish are apparent. Populations of mesopredators started to decrease after stocking of catfish and their abundances were lower than abundances of omnivores in most of the monitored years (Most, Milada). I n contrast, marked increase of abundances of mesopredators with time was observed in the reference lake (Medard). Their abundances significantly exceeded the abundances of omnivores with time. The fundamental impact of apex predators on mesopredators is well known^15^. The impact is induced by i) direct competition for food or territory^3,15^, ii) killing of weaker mesopredators as unwanted competitors^3^, and also iii) hunting for mesopredators as a food source^3^. In the case of European catfish, the latter impact seems to be the most relevant, because preference for mesopredators is low (−0.33 and −0.08 for Most and Milada, respectively) and hence the mesopredators are not killed to get rid of competitors. In spite of the low presence of mesopredators in catfish diet, decrease of their abundances is caused by their lower resistance to the predation pressure in comparison to that of omnivores. The catfish theoretically consume only 17.1–39.8 kg and 20.1–46.9 kg of perch per year in Most and Milada, respectively. However, the predation pressure on perch is raised by the consumption of their egg strands^28^. The predation pressure on mesopredators is sufficient to reduce their biomass to a tolerable level in the ecosystem that consequently stagnates, therefore mesopredators are able to coexist with the European catfish. We are well aware of differences in life cycles and strategies between freshwater and terrestrial predators, but similar coexistence relationships may be observed in several terrestrial predators from the order carnivora^4^.

Regarding human management in aquatic ecosystems with natural occurrence of both European catfish and Northern pike, it is advisable to maintain the coexistence of both species. Multiple predator effects provide high stability and well-balanced biodiversity in the ecosystem^43^. Cannibalism in European catfish was proved in both lakes, but it was quite a rare phenomenon. Thus catfish is a less cannibalistic species than pike or perch^32,34^. It probably occurs only for the purpose of food intake, not as an evolutionary strategy. This strategy is often observed in the order Carnivora where cannibalism or even infanticide is connected with an attempt to increase their fitness and thus to decrease the fitness of an intraspecific competitor^44^.

The high contribution of herbivores (mainly rudd) in catfish diet was a surprising finding, considering their relatively low biomass in the lakes. In addition, rudd was definitely a preferred food item. The nocturnal activity of these potential preys is presumably the main reason (see the description above). In terms of rudd, predation occurs also during the day due to its inefficient antipredation behaviour^39^. Similarly, a fundamental part of the diet of terrestrial apex predators is composed of herbivores. It is closely linked to the key impact of apex predators on ecosystem structure, that is commonly shaped by herbivores^3,11^. The cascade effect, called top down effect, has been documented in the freshwater ecosystem long before^45^, and this effect leads to a final impact on phytoplankton as a primary producer. Whether an apex predator may also affect species richness and cover of macrophytes, the main food item of aquatic herbivores^46^, has still not been answered. Nevertheless, our preliminary results indicate this mentioned effect (Vejříková, in prep.). Due to insufficient comparison of herbivorous rudd in lakes with and without catfish, we cannot make confident conclusions on the impact of catfish on its population. However, substantial impact is very probable due to the strong preference of rudd in catfish diet, high number of rudd individuals consumed every year in both study lakes (see Table 2) and apparent decrease of rudd abundances in both lakes after catfish stocking.

Although predator-prey interactions depend on density of prey^47^, the most numerous species, roach, was avoided in the diet for most of the year. It is probably due to roach sleeping at night (SCUBA observations; L. Vejřík, J. Peterka, M. Čech, unpubl. data) and various antipredation mechanisms during the day^39^. In contrast, a strong preference for roach in catfish diet near its spawning area during reproduction in Most Lake clearly illustrates the rapid ability of European catfish to learn and directly utilize a new, easily available food source. During reproduction, cyprinids gather at spawning areas in abundant shoals and their alarm cues (chemical substance released from injured fish skin that provides warning for surviving individuals) are suppressed^48^. Risk perception is generally lower for all animals during reproduction^49^. European catfish were also observed close to spawning areas of rudd in Milada Lake (F. Uhlíř, pers. comm.) and of bream in Římov Reservoir (J. Seďa, pers. comm.). Therefore, visiting and hunting on cyprinids spawning grounds seems to be a common and very efficient strategy for catfish to meet their dietary needs. Their great ability to adapt to currently available food sources is also proved by the occurrence of perch egg strands in the diet^28^.

Macrophytes were the most unexpected food item found in the catfish stomach. It composed 7% of total diet biomass in Milada Lake. Plants have already been recorded in catfish diet^23^, nevertheless, accidental ingestion of these items during suction of benthic prey (larvae of dragonflies, crayfish etc.) is a more probable explanation for the presence of macrophytes in catfish stomachs than intentional feeding on them. The question is whether ingested plant material may provide energy to the catfish. The fish digestive tract works differently than that of higher vertebrates. Tracts of closely related carnivorous and herbivorous fish may be very similar but differ in digestive biochemistry that plays a key role^50^. Microorganisms responsible for digestion of plant material get into the tract mainly from detritus^46^. Hence, we may assume that European catfish also has the potential to digest plants. This presumption is supported by videos recorded in Chernobyl cooling pond, where European catfish are intensively fed with bread (check YouTube: www.youtube.com/watch?v=3cEj8R5m3AI; www.youtube.com/watch?v=qf7n2kLubUQ and others). It sheds new light on the generalist behaviour of this apex predator. In relation to habitat, the dietary niche of European catfish extends from primarily marine food sources^26^, to terrestrial prey^27^, semiaquatic prey and freshwater prey across clades of freshwater animals^23^ and plant material.

An analogical apex predator in a terrestrial ecosystem would be the grizzly bear (*Ursus arctos horribilis*). It is also a true generalist with a similarly wide diet spectrum and short-term individual specialization^51^. In the marine environment, the most similar generalist would probably be the tiger shark^10^. Unusually wide niche of catfish is also proved by extra-large PPR (0.04–0.51 and 0.05–0.57 for Most and Milada Lakes, respectively) in contrast to narrow PPR of Northern pike (0.14–0.29 and 0.15–0.30) and PPR of other freshwater predators^52^ and related studies.

The relatively high number of European catfish individuals with empty stomachs (40 and 72%) is not surprising, because the strategy “run on empty” is very common for predators^53^. Common frequency of empty stomachs of catfish is 20–78%^23^. In case of Most where the estimated mean annual mass increase of catfish is c. 381 g, sufficient amount of food for catfish specialized on waterfowl (*i*.*e*. abnormal prey in size) seems to be two or three individuals of waterfowl per year. In Milada with estimated mean annual mass increase of catfish c. 1.1 kg, six individuals of waterfowl should be sufficient. However, mean mass of the prey consumed in Most and Milada was 269 and 242 g, respectively. Individuals would have to utilize eight and 25 of these average preys per year in Most and Milada, respectively. Although, such irregular food intake has not been sufficiently recorded among fish, it is a common phenomenon found among aquatic apex predators from ectothermic vertebrates^54^. Nevertheless, the reason of such high occurrence of catfish with empty stomachs and such high frequency of our baits in the stomachs in Most is also caused by the low food availability in the lake. It is also evident from the low trophic status of the lake. The distinctly lower degree of IS for European catfish in Most Lake, which signifies high individual niche specialization (INS), is probably induced by higher intraspecific and conversely lower interspecific competition^12^. The other reasons are i) biomass of catfish is 28% higher in Most than Milada, ii) the biomass of pike is according to catches much lower in Most than Milada, and iii) the other mesopredator, asp, is totally absent in Most Lake^55^.

WIC and TNW of pike are distinctively lower than that of catfish. As mentioned above, pike prefers only a few prey species. Its partial utilization of semiaquatic prey in Most Lake is presumably due to low food availability in this oligotrophic lake. In contrast, the stomach contents of catfish indicate the true generalist behaviour of this species. However, SIA showed marked differences among individuals. It proves that the generalist population contains many specialist individuals, specializing on semiaquatic or terrestrial prey^27^. Similarly, the population of alligator (*Alligator mississippiensis*), the main apex predator of freshwaters in North America, is composed of both generalist and specialist individuals, specializing on difficult to catch prey^7^. INS aimed at large and high-energy prey is an advantageous target for apex predators. The learned ability to utilize one type of large prey^13^ considerably shortens handling time^56^. This is probably the reason why some individuals from our lakes focused on semiaquatic prey. Catching and handling of this type of prey is difficult and learning how to do it is more complicated and challenging in comparison to utilization of aquatic prey^11,27^.

According to WIC and seasonal preferences of certain food sources, the majority of catfish individuals seem to have high short-term INS. This phenomenon for apex predators has already been described^7^. Hence the widely accepted paradigm stating that generalist populations are composed of specialist individuals^12^ may be in some cases biased by short-term specialist individuals that appear to be long-term generalist individuals when the whole year is taken into consideration. The effects of prey composition on short-term INS are particularly important to investigate for large apex predators. They generally move long distances and thus inhabit various ecosystems with different types of prey^7^. Hence apex predators need to be ready to turn a profit from a new niche and utilize food sources that were until recently unfamiliar to them. This situation may be observed in killer whales (*Orcinus orca*) that, besides forming a highly specialized population across the world, are also able to switch to a different food source when the preferred source is absent^14^. In addition, killer whales are spreading to polar regions due to climate changes, where they utilize completely new food sources^6^. The ability to learn efficiently and become specialized on newly available prey^13^ plays a key role in becoming a successful apex predator for these reasons i) it is probably the most efficient method to satisfy the energy requirements of a large body, and ii) this ability enables the maintenance of a relatively numerous population of the apex predator species. However, nowadays the second reason mostly cannot be realized because of the negative impact of humans^2^. This scenario is not true for European catfish thanks to its popularity among anglers^23^.

Comparison of fish community in the lakes with catfish (Most and Milada) and without catfish (Medard) shows that catfish markedly affect the ecosystems. Although the study and reference lakes have very similar characteristics, uncertain distinctness should be also taken into account in the fish community development. However, catfish had a great impact on the populations of rudd and mesopredators. The total amount of consumed food per year calculated using annual mass increase of catfish and feed conversion ratio (FCR)^57^ is 530.9–1,238.7 kg and 487.3–1,137.8 kg for Most and Milada, respectively. The values in the upper limits are more probable considering the low trophy of the lakes and necessarily longer time for searching the prey. Then, catfish population annually utilize 3.7 kg and 5 kg of prey per 1 hectare of Most and Milada, respectively. In case of fish prey (excluding cannibalism), it is 2.2 kg and 3.8 kg per 1 ha of Most and Milada, respectively. Fish biomass (excluding catfish) in 2013–2015 was estimated at 11.3–27.5 kg and 14.9–28.3 kg per 1 ha of Most and Milada, respectively^55,58^. Thus the fish preys consumed by catfish make 8–20% and 13–26% of total fish biomass in Most and Milada, respectively. However, the values are not totally accurate and have only information character.

### Conclusion and recommendation for future studies

The European catfish niche width widely exceeds the niche width of Northern pike, the second biggest fish predator in Europe. In the presence of catfish, pike fulfils the role of mesopredator. Nevertheless, the position of pike is still irreplaceable due to multiple predator effects.

European catfish has many behavioural features in common with other apex predators^2^. Catfish is raised to the position of successful apex predator thanks to features such as wide diet plasticity and good adaptability to new food sources^26–28^ associated with distribution of various food sources among individuals. These features, in conjunction with a low level of cannibalism, allow catfish to establish highly abundant populations^23^. This is the reason why European catfish is an ideal species to study the impact of an apex predator in an ecosystem in real time. Further, thanks to its human-mediated spread to new localities (particularly by anglers), future studies may focus on continuous changes in communities from all trophic levels in the ecosystem, such as phytoplankton or macrophytes.

Broad adaptability and learning ability to utilize various food sources may be an important feature of the trophic dynamics of an apex predator. It should be considered in studies focused on freshwater food webs and the ecological role of apex predators.

As far as we know, our study is based on the largest dataset collected in natural conditions among studies focused on the diet of European catfish. Hence, we would recommend the spread of gained information among the public, particularly among anglers, to avoid inaccurate conclusions concerning the dietary behaviour of catfish.

## Methods

### Study site

**Figure 8:**
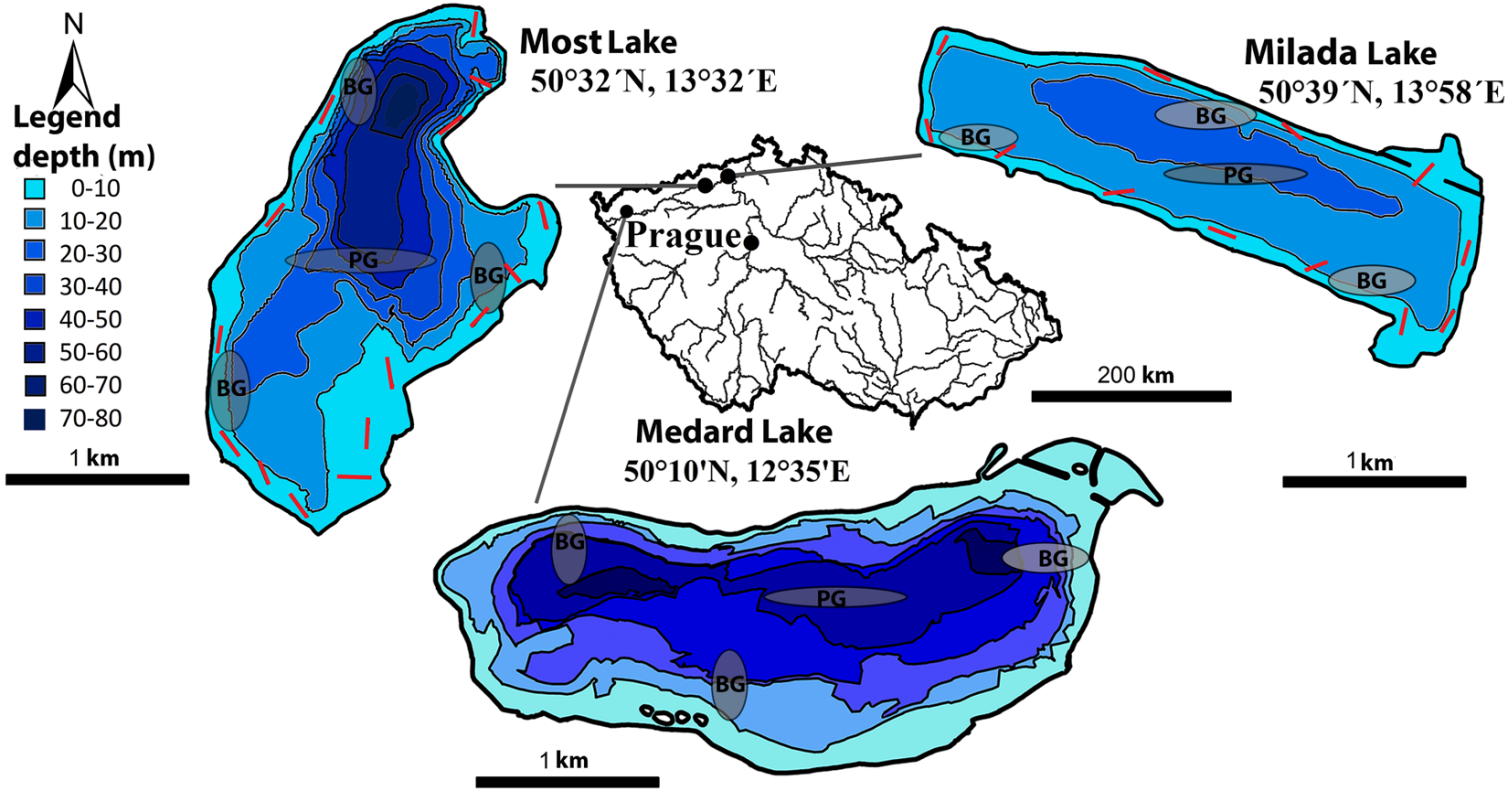
Map showing the location and relevant depths of the two study lakes, Most and Milada, and the reference Medard Lake, Czech Republic. Localities sampled by longlines are shown by red lines along the lake shores, and localities sampled by gillnets by grey ellipses with BG and PG for benthic and pelagic gillnets, respectively. The figure was generated by the software ArcMap, version 10.2.2^65^.

The study was conducted in two lakes created after aquatic restorations of mining pits, Most and Milada Lakes, Czech Republic. The oligotrophic Most Lake has an area of 310 ha, volume of 70 × 10^6^ m^3^ and maximum depth of 75 m, and the oligo to mesotrophic Milada has an area of 250 ha, volume of 36 × 10^6^ m^3^ and max. depth of 25 m (Fig. 8). Aquatic restoration in Most lasted six years (2008–2014) and in Milada ten years (2001 to 2011). Fish community is similar in both lakes. Fishes occurring already in retention pool of the mining pit were rudd (herbivore), roach, ruffe, tench (omnivores) perch (mesopredator) in both lakes, and rarely asp and pikeperch (*Sander lucioperca*) (mesopredators) in Milada Lake. Whitefish (*Coregonus sp*., omnivorous fish) was introduced to Most Lake in 2011–2013^46^. European catfish (apex consumer) and Northern pike (mesopredator) were stocked for biomanipulation purposes because absence of large predators raised concerns about expansion of zooplanktivorous fish that may have a negative impact on the water quality. In Most, Northern pike (2,332 individuals, mean mass 1.1 kg) and European catfish (694 individuals, mean mass 3.7 kg) were both introduced in 2011, 2012 and 2013. In Milada, Northern pike was introduced in 2005 (789 individuals, mean mass 0.3 kg) and low number of small European catfish in 2005 (12 individuals, mean mass 7.7 kg) but the catfish was introduced mainly in 2007 (316 individuals, mean mass 1.2 kg), In both lakes, all introduced catfish and pike were individually tagged with a PIT-tag (passive integrated transponder tag, Oregon RFID, full-duplex, length 12 mm, diameter 2.15 mm, mass 0.11 g, 11784/11785 compatible).

The reference lake, Medard, was also created after aquatic restorations of mining pits. It is an oligotrophic lake with an area of 493 ha, volume of 50 × 10^6^ m^3^ and maximum depth of 55 m. Aquatic restoration lasted eight years (2008–2016). Fish community occurring already in retention pool of the mining pit was composed of roach, ruffe (omnivorous fish) and Northern pike (mesopredator). In contrast to Most and Milada, pike occurred from the beginning of the water restoration. During water filling in 2012–2014, new species came from the river: perch, pikeperch (mesopredators) tench, European chub (*Squalius cephalus*), common bream (*Abramis brama*) and silver bream (*Blicca bjoerkna*) (omnivorous fish). In 2015, rudd (herbivorous fish) occurred in the lake. Whitefish (omnivorous fish) were introduced to Medard Lake in year 2012-2014. Catfish was not stocked to the lake (J. Peterka, unpubl. data).

### Fish sampling and stomach content analysis

Animal treatment was performed in accordance with guidelines from the Experimental Animal Welfare Commission under the Ministry of Agriculture of the Czech Republic (Ref. No. CZ 01679) and with permission of the owners of the study sites, Palivový kombinát Ústí, státní podnik (Most, Milada) and Sokolovská uhelná a.s. (Medard). The Experimental Animal Welfare Commission approved all experimental protocols.

European catfish and Northern pike from both lakes were caught by longlines from August 2013 to May 2015 always during a 4-day-and-night-long campaign. Eight campaigns were conducted on each lake from spring to late autumn (May to November), i.e. 32 days in each lake. See^28^ for details about the longline method. Three lines, each with 10 bait fish, were used and were moved every day of sampling to a new place to cover the shore evenly. They were checked three times per day (before dusk, soon after midnight, and shortly after dawn). Each caught predatory fish was measured, weighed, a small part of fin was cut for SIA and non-invasive stomach content analysis was provided^46^. Stomach content of catfish was extracted by hand through opened mouth and gullet. Stomach content of pike was washed out through a larger tube into a jar, while water was pumped through a small tube into the pike’s stomach. The latter method was not fully effective as we tried to prevent any harm to fish. All fish were released back into the lake as soon as possible. The stomach contents were subsequently identified, or fixed with 70% ethanol for laboratory identification using diagnostic elements such as fish bones see [S1^59^,]. Only fish stocked at least 660 days ago were used for SIA due to turnover of isotopic signal^10^.

Prey fishes were sampled in September 2011–2015 in Most and 2005–2015 in Milada by both benthic and pelagic multi-mesh gillnets in both lakes for details see^46^. 36 gillnets (24 benthic and 12 pelagic) were set in each lake and year, summing to a total of 144 gillnet nights and 8,640 m^2^ gillnet area. Fish biomass (kg of fish > 0 + per 1,000 m^2^ of gillnets) in Fig. 7 was calculated as the weighted average of each depth zone using both littoral and pelagic gillnets. In total, 4,876 and 6,753 individuals of eight fish species found in catfish diet were captured in Most and Milada, respectively. Prey samples for SIA were sampled in 2014, it means the same year as the most of the predators.

All captured fish were immediately anaesthetized by a lethal dose of tricainemethanesulfonate (MS–222, Sigma Aldrich Co.). Mammals, birds, amphibians and large-sized benthic odonates were collected during campaigns of catfish sampling. From each fish (randomly chosen fish individuals of all potential prey species) and other potential prey sources, a small piece of muscle tissue was dissected and stored frozen at −20 °C prior to final preparation for SIA. Six to eight replications of each prey sample found in catfish stomachs were used for SIA.

### Size of captured individuals, time period of the catch and mass increase of recaptures

Altogether we captured 232 and 93 catfish individuals in Most and Milada, respectively. Out of these individuals, there were 74 and 37 recaptures in Most and Milada, respectively. Mean size and mean mass were 85 cm and 4.1 kg (min: 55 cm and 0.65 kg, max: 128 cm and 11.8 kg) in Most Lake, and 103 cm and 8.4 kg (min: 67 cm and 2.2 kg, max: 158 cm and 23.5 kg) in Milada Lake. The majority of individuals (87%) were captured during the night. All captured individuals were stocked and tagged. Individuals born in the lakes did not reach the sizes of individuals that are caught by long-lines (L_T_ > 70 cm), moreover, due to the predation pressure and competition they are extremely scarce in both lakes (J. Peterka, unpubl. data). Annual mass increase of recaptured individuals was approximately 381 g and 1,100 g in Most and Milada, respectively.

We captured 18 and 84 pike individuals in Most and Milada, respectively. Out of these individuals, there were 2 and 10 recaptures in Most and Milada, respectively. Mean size and mean mass were 84 cm and 4.2 kg (min: 69 cm and 1.8 kg; max: 97 cm and 6.1 kg) in Most Lake, and 84 cm and 5.1 kg (min: 48 cm and 0.55 kg, max: 120 cm and 14.3 kg) in Milada Lake. All individuals were captured during the day. All pikes from Most Lake were stocked and tagged. In Milada Lake, 28% of captured pikes were tagged, other individuals originated from the natural reproduction in the lake.

### Stable Isotope Analysis

All frozen SIA samples were later dried at 60 °C for 48 h and ground into a homogenous powder using a ball-mill Retsch MM 200 (Retsch GmbH, Haan, Germany). Small subsamples (0.52–0.77 mg) were weighed into tin cups for the analysis of δ^13^C and δ^15^N. All SIA were conducted using a FlashEA 1112 elemental analyser coupled to a Finnigan DELTA^plus^ Advantage mass spectrometer (Thermo Fisher Scientific Corporation, Waltham, MA, U.S.A.) at the University of Jyvaskyla, Finland. Stable nitrogen and carbon isotope ratios are expressed as δ^15^N and δ^13^C relative to the international standards for nitrogen (atmospheric nitrogen) and carbon (Vienna PeeDeeBelemnite). Analytical precision was ± 0.20‰ for both isotopes, and was determined by repeated analysis of a working standard (pike white muscle tissue) inserted in each run after every five samples. As C:N ratios were consistently lower than 3.5 (i.e., > 90% of cases), obtained stable isotope values of fish were not lipid corrected^60^.

### Statistical analysis

The SIAR package (Stable Isotope Analysis in R; version 4.2;^35^) was used to estimate the relative contributions of semiaquatic and aquatic prey in the long-term diets of catfish and pike. The SIAR input data included individual δ^13^C and δ^15^N values from catfish and pike fin, mean ± SD δ^13^C and δ^15^N values of muscle tissue from potential semiaquatic and aquatic prey sources, and the commonly used trophic fractionation corrections of 0.4 ± 1.3‰ for δ^13^C and 3.4 ± 1.0‰ for δ^15^N^61^. Finally, the SIBER package (Stable Isotope Bayesian Ellipses in R; version 2.0.3;^36^) was used to estimate sample-size corrected standard ellipse areas (*SEA*_c_), total convex hull areas (*TA*), and proportional overlap of the *SEA*_c_ areas. The estimated *SEA*_c_ and *TA* areas indicate the long-term dietary niche widths of catfish and pike, whereas the proportional overlap between *SEA*_c_ areas measures the degree of dietary niche segregation between the two predatory fishes. For SIAR and SIBER analyses, 74 and 69 of catfish samples, and 18 and 32 of pike samples were used from Most and Milada, respectively. No recaptures were used in SIAR and SIBER analyses and both were done in R 3.1.1^62^.

Trophic specialization is calculated as dietary variation within individuals (WIC: within individual component of variation) and between individuals (BIC: between individual component of variation) of a population. The WIC of a population measures how variable an individual’s diet is over time period. It is typically expressed as a mean value for an entire population, but can be similarly assessed for individuals. It was calculated from 16 and 16 recaptured catfish, and 2 and 8 recaptured pikes from Most and Milada, respectively. The BIC of a population measures how different each individual’s diet is from the other members of the population^63^. Low values of WIC indicate individuals and populations that are more specialized, as individual diets show little variation and should be consistent over time, and vice versa^63^. The BIC varies based on total niche width (TNW). IS (degree of individual specialization) is calculated as WIC/TNW ratio and reaches values from 0 to 1. A high value means that all individuals utilize the entire niche of the species, whereas low values signify low intraspecific overlap and thus greater individual niche specialization (INS)^10^. WIC, BIC, TNW and degree of IS were calculated from δ^13^C in the Ind Spec1 program^64^.

The prey-to-predator length ratio (PPR) was calculated as:

$$PPR={L}_{T}Py/{L}_{T}Pr$$

where *L*_*T*_ *Py* represents *L*_*T*_ (total length) of prey and *L*_*T*_ *Pr* represents *L*_*T*_ of a predator^52^. Perch egg strands and macrophytes were excluded from the calculations, whereas fish baits, that were found in the stomachs, were included. In total, 101 and 106 diet samples from catfish stomachs, and 6 and 12 diet samples from pike stomachs were used from Most and Milada, respectively.

Electivity index, *E*_*i*_ defined for a group (*i*) as:

$${E}_{i}=({r}_{i}-{P}_{i})/({r}_{i}+{P}_{i})$$

where *r*_*i*_ represents the relative biomass of a prey in a predator’s diet and *P*_*i*_ represents the prey’s relative biomass in the ecosystem. *E*_*i*_ = −1 means total avoidance of, *E*_*i*_ = 0 means non-selective feeding on, and *E*_*i*_ = 1 means exclusive feeding on a given prey *i*.

Estimated size of the catfish biomass from stocking to 2015 shown in Fig. 7 was calculated from stocked biomass in the years 2011–2013 for Most, and 2005 and 2007 for Milada. Gillnet sampling in a given year was always conducted prior to the stocking of predators, thus biomass and potential impact of fish predators on fish communities was considered for the following year. Estimated size of population for Most in 2015 and for Milada in 2014–2015 was calculated from recaptures (Vejřík unpubl. data). Year 2011 in Milada, when sampling was not conducted, was spaced with a straight line. The size of pike biomass was not estimated due to an insufficient number of recaptures. Fish biomass (kg per 1,000 m^2^ of gillnets) in Fig. 7 was calculated as the weighted average for each depth zone using both littoral and pelagic gillnets. A chi-square test (χ^2^) was used to compare the contribution of each food item among the seasons. For the analyses, we used 48 and 53 fish samples found in catfish stomachs from Most and Milada, respectively.

Total consumption (TC) of food utilized by catfish was calculated as:

$$TC=N\ast FCR$$

where *FCR* represents feed conversion ratio showing total biomass of each food item utilized annually, and ranges from 2.4 to 5.6 kg of food per 1 kg of mass increase in natural ecosystems^57^ and related studies. *N* represents total number of adult catfish and was estimated (using recaptures) at 577 and 180 individuals in the populations of Most and Milada, respectively^28^. The contribution of each food item was then calculated from the percentage ratio of all food items found in catfish stomachs, see Fig. 2A. Differences in nutrition values were not taken into account.

### Data availability

All data analysed during this study are included in this published article (and its Supplementary Information files).

### Ethics

Animal treatment was performed in accordance with guidelines from the Experimental Animal Welfare Commission under the Ministry of Agriculture of the Czech Republic (Ref. No. CZ 01679) and with permission of the owner of the study sites, Palivový kombinát Ústí, státní podnik. The Experimental Animal Welfare Commission approved all experimental protocols.

## Electronic supplementary material

Table S1

Dataset
