# Supplementary material for: European catfish (Silurus glanis) as a freshwater apex predator drives ecosystem via its diet adaptability
Source: Sci Rep. 2017 Nov 21;7:15970. doi: 10.1038/s41598-017-16169-9 (PMC5698325; doi:10.1038/s41598-017-16169-9)
Supplement: Supplementary file 1 — Table S1 [file 41598_2017_16169_MOESM1_ESM.pdf]

European catfish (*Silurus glanis*) as a freshwater apex predator drives ecosystem via its diet adaptability

Lukáš Vejřík[1,2*], Ivana Vejříková[1], Petr Blabolil[1], Antti P. Eloranta[3], Luboš Kočvara[1], Jiří Peterka[1], Zuzana Sajdlová[1], Son Chung Hoang The[1], Marek Šmejkal[1],Mikko Kiljunen[4], Martin Čech[1]

[1]Biology Centre of the Czech Academy of Sciences, Institute of Hydrobiology, Na Sádkách 7, 37005 České Budějovice, Czech Republic

[2]Faculty of Science, University of South Bohemia in České Budějovice, Branišovská 31, 37005 České Budějovice, Czech Republic

[3] Norwegian Institute for Nature Research, P.O. Box 5685 Sluppen, NO-7485 Trondheim Norway

[4] University of Jyväskylä, Department of Biological and Environmental Science, P.O. Box 35, FI-40014 University of Jyväskylä, Finland

Correspondence to: vejrik.lukas@seznam.cz

Table S1. Regression equations for back-calculation of total length (L_T; cm) of individual prey fish species from recovered diagnostic bones (bone dimension in mm). PhT=pharyngeal tip, DeL=dental length, MxL=maxilar length, PpL=preopercular length and PpG=preopercular gape.

Family / species	Bone measurement	Equation, L_T=	Reference*
Cyprinidae			
Roach *Rutilus rutilus*	PhT	1.5658PhT+0.2805	Čech & Vejřík (2011)
Rudd *Scardinius erythrophthalmus*	PhT	1.4894PhT+0.6513	Čech et al. (2008)
Gudgeon *Gobio gobio*	PhT	1.9278PhT–0.0653	Čech & Vejřík (2011)
European Chub *Squalius cephalus*	PhT	1.3733PhT–0.025	Čech et al. (2008)
Common Dace *Leuciscus leuciscus*	PhT	1.8579PhT–0.9119	Čech & Vejřík (2011)
Bleak *Alburnus alburnus*	PhT	2.1733PhT+0.1657	Čech et al. (2008)
Belica *Leucaspius delineatus*	PhT	1.6855PhT+0.1111	Čech & Čech (2013)
Asp *Aspius aspius*	PhT	1.4942PhT+1.1551	Čech et al. (2008)
Common Bream *Abramis brama*	PhT	1.7671PhT+0.244	Čech et al. (2008)
White Bream *Blicca bjoerkna*	PhT	1.7206PhT+1.7419	Čech et al. (2008)
Nase *Chondrostoma nasus*	PhT	1.9032PhT+2.3628	M. Čech, unpubl.data
Tench *Tinca tinca*	PhT	1.3262PhT–0.3947	Čech et al. (2008)
Common Carp *Cyprinus carpio*	PhT	1.1102PhT–2.8219	Čech et al. (2008)
Prussian Carp *Carassius auratus*	PhT	1.0521PhT+0.9672	Čech & Čech (2017)
Stone Moroko *Pseudorasbora parva*	PhT	2.3262PhT–0.2566	Čech & Čech (2013)
Eurasian minnow *Phoxinus phoxinus*	PhT	1.5703PhT+0.5619	M. Čech, unpubl.data
Anquillidae			
European eel *Anguilla anguilla*	DeL	1.7691DeL+8.8001	Čech & Vejřík (2011)
Salmonidae			
Trout spp.	MxL	1.0755MxL+1.8713	Čech & Vejřík (2011)
	DeL	1.3191DeL+2.0274	Čech & Vejřík (2011)
Esocidae			
Northern Pike *Esox lucius*	DeL	0.6981DeL+3.9125	Čech et al. (2008)
Cottidae			
Bullhead *Cottus gobio*	PpL	1.203PpL–0.9609	Čech & Vejřík (2011)

Family / species	Bone measuremen t	Equation, L_T=	Reference
Percidae			
European Perch *Perca fluviatilis*	DeL	1.1217DeL+1.7753	Čech et al. (2008)
	PpG	0.8821PpG+0.9797	Čech et al. (2008)
Ruffe *Gymnocephalus cernuus*	DeL	1.6758DeL–0.1998	Čech et al. (2008)
	PpG	0.7923PpG+0.4587	Čech et al. (2008)
Zander *Sander lucioperca*	DeL	1.0821DeL–1.17	Čech et al. (2008)
	PpG	1.1815PpG–1.2726	Čech et al. (2008)

*For the methods of measurement, see the following studies: 1) Čech, M. & Čech, P. Effect of brood size on food provisioning rate in Common Kingfishers *Alcedo atthis*. *Ardea* (2017, in press). 2) Čech, M. & Čech, P. The role of floods in the lives of fish-eating birds: Predator loss or benefit? *Hydrobiologia* 717(1), 203-211 (2013). 3) Čech, M. & Vejřík, L. Winter diet of great cormorant (*Phalacrocorax carbo*) on the River Vltava: estimate of size and species composition and potential for fish stock losses. *Folia Zool*. 60, 129–142 (2011). 4) Čech, M., Čech, P., Kubečka, J., Prchalová, M. & Draštík, V. Size selectivity in summer and winter diets of great cormorant (*Phalacrocorax carbo*): Does it reflect season-dependent difference in foraging efficiency? *Waterbirds* 31(3): 438-447 (2008).
